# 11-[(*E*)-Benzyl­idene]-14-hy­droxy-8-phenyl-6-thia-3,13-diaza­hepta­cyclo­[13.7.1.1^9,13^.0^2,9^.0^2,14^.0^3,7^.0^19,23^]tetra­cosa-1(22),15(23),16,18,20-pentaen-10-one

**DOI:** 10.1107/S1600536812024270

**Published:** 2012-06-13

**Authors:** Raju Suresh Kumar, Hasnah Osman, Abdulrahman I. Almansour, Suhana Arshad, Ibrahim Abdul Razak

**Affiliations:** a School of Chemical Sciences, Universiti Sains Malaysia, 11800 USM, Penang, Malaysia; b Department of Chemistry, College of Sciences, King Saud University, PO Box 2455, Riyadh 11451, Saudi Arabia; c School of Physics, Universiti Sains Malaysia, 11800 USM, Penang, Malaysia

## Abstract

In the title compound, C_34_H_28_N_2_O_2_S, the piperidine ring adopts a chair conformation. One of the pyrrolidine rings adopts an envelope conformation with the methyl­ene C atom at the flap whereas the other pyrrolidine ring and the thia­zolidine ring adopt half-chair conformations. The mean plane of the dihydro­acenaphthyl­ene ring system [maximum deviation = 0.067 (1) Å] makes dihedral angles of 28.31 (5) and 31.32 (6)° with the two terminal benzene rings. An intra­molecular O—H⋯N hydrogen bond forms an *S*(5) ring motif. In the crystal, mol­ecules are linked by C—H⋯O and C—H⋯S hydrogen bonds into layers lying parallel to the *ac* plane.

## Related literature

For general background to heterocycles, see: Corey *et al.* (2007 ▶); Padwa (1984 ▶); Lee *et al.* (2001 ▶); Lalezari & Schwartz (1988 ▶); Aicher *et al.* (1998 ▶). For related structures, see: Kumar *et al.* (2010*a* ▶,*b* ▶, 2011 ▶). For ring conformations, see: Cremer & Pople (1975 ▶). For hydrogen-bond motifs, see: Bernstein *et al.* (1995 ▶). For the stability of the temperature controller used in the data collection, see: Cosier & Glazer (1986 ▶).
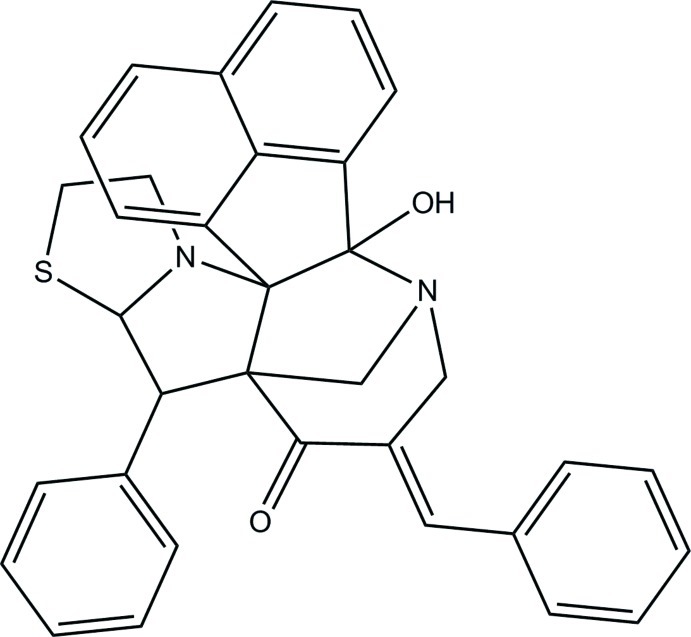


## Experimental

### 

#### Crystal data

C_34_H_28_N_2_O_2_S
*M* *_r_* = 528.64
Monoclinic, 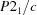

*a* = 11.2911 (1) Å
*b* = 15.4317 (2) Å
*c* = 15.1920 (2) Å
β = 92.790 (1)°
*V* = 2643.93 (5) Å^3^
*Z* = 4
Mo *K*α radiation
μ = 0.16 mm^−1^
*T* = 100 K
0.45 × 0.41 × 0.31 mm

#### Data collection

Bruker SMART APEXII CCD diffractometer
Absorption correction: multi-scan (*SADABS*; Bruker, 2009 ▶) *T*_min_ = 0.932, *T*_max_ = 0.953
37231 measured reflections
9686 independent reflections
8170 reflections with *I* > 2σ(*I*)
*R*_int_ = 0.025

#### Refinement

*R*[*F* ^2^ > 2σ(*F* ^2^)] = 0.046
*wR*(*F* ^2^) = 0.129
*S* = 1.04
9686 reflections
344 parameters
H atoms treated by a mixture of independent and constrained refinement
Δρ_max_ = 1.18 e Å^−3^
Δρ_min_ = −1.12 e Å^−3^

### 

Data collection: *APEX2* (Bruker, 2009 ▶); cell refinement: *SAINT* (Bruker, 2009 ▶); data reduction: *SAINT*; program(s) used to solve structure: *SHELXTL* (Sheldrick, 2008 ▶); program(s) used to refine structure: *SHELXTL*; molecular graphics: *SHELXTL*; software used to prepare material for publication: *SHELXTL* and *PLATON* (Spek, 2009 ▶).

## Supplementary Material

Crystal structure: contains datablock(s) global, I. DOI: 10.1107/S1600536812024270/hb6800sup1.cif

Structure factors: contains datablock(s) I. DOI: 10.1107/S1600536812024270/hb6800Isup2.hkl

Supplementary material file. DOI: 10.1107/S1600536812024270/hb6800Isup3.cml

Additional supplementary materials:  crystallographic information; 3D view; checkCIF report

## Figures and Tables

**Table 1:** Hydrogen-bond geometry (Å, °)

*D*—H⋯*A*	*D*—H	H⋯*A*	*D*⋯*A*	*D*—H⋯*A*
O2—H1*O*2⋯N2	0.86 (2)	1.95 (2)	2.6277 (12)	134.4 (18)
C8—H8*A*⋯O1^i^	0.99	2.55	3.1981 (14)	123
C15—H15*A*⋯S1^ii^	0.95	2.72	3.4970 (13)	139
C18—H18*A*⋯O1^iii^	0.95	2.60	3.2550 (15)	127
C24—H24*A*⋯O2^iv^	0.95	2.59	3.4077 (18)	145

Symmetry codes: (i) 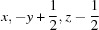; (ii) 
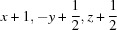
; (iii) 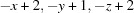; (iv) 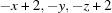.

**Fig. 1.**
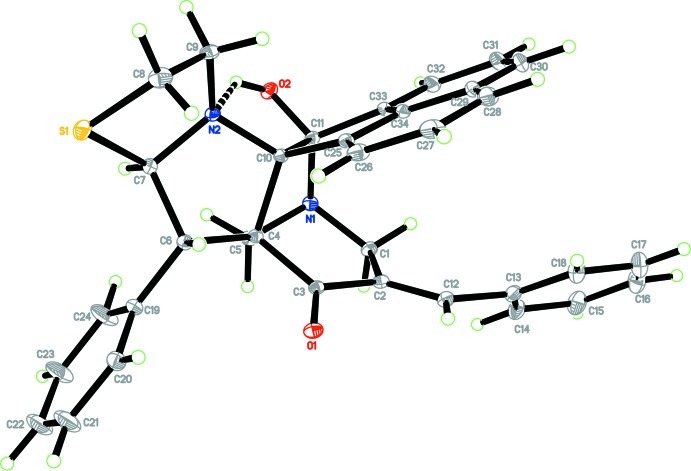
The molecular structure of the title compound, showing 30% probability displacement ellipsoids.

**Fig. 2.**
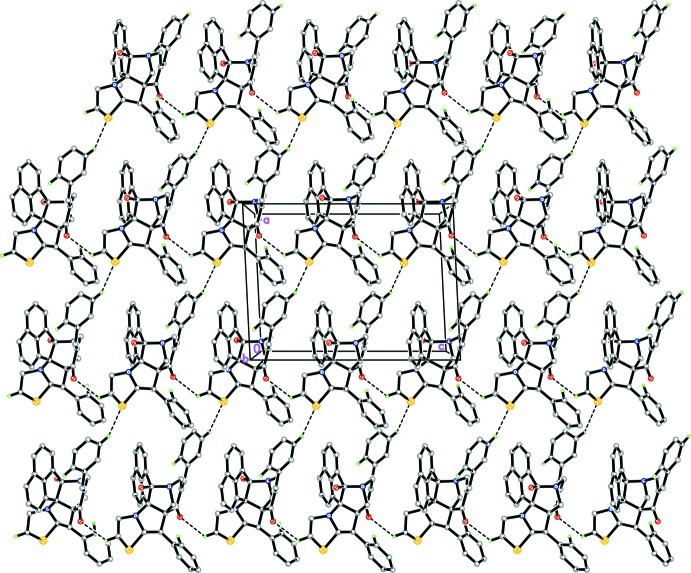
The crystal packing of the title compound. The H atoms not involved in the intermolecular interactions (dashed lines) have been omitted for clarity.

## supplementary crystallographic information

### Comment

The synthesis and chemistry of heterocyclic compounds has been an interesting field in view of their structural diversity and remarkable ability to serve as biomimetics and active pharmacophores. Many of the most famous natural alkaloids or unnatural drugs consist of at least one heterocyclic ring (Corey *et al.*, 2007). 1,3-Dipolar cycloaddition of azomethine ylides to olefinic dipolarophiles affords five membered heterocyclic rings of biological importance (Padwa, 1984). Heterocycles with piperidine sub-structures are being used as synthons in the construction of alkaloid natural products (Lee *et al.*, 2001). Pyrrolothiazole ring possesses antineoplastic (Lalezari & Schwartz, 1988) and hypoglycemic (Aicher *et al.*, 1998) activity. The importance of aforesaid heterocycles, incited us to investigate the X-ray diffraction study of the title compound and report the results in this paper.

In the molecular structure (Fig. 1), the piperidine ring (N1/C1–C5) adopts a chair conformation with puckering parameters (Cremer & Pople, 1975), Q= 0.6090 (10) Å, Θ= 37.98 (10)° and Φ= 303.45 (16)°.

For the two pyrrolidine rings, N1/C4/C5/C10/C11 is in envelope conformation with atom C5 deviating by 0.298 (1) Å from the mean plane through the remaining atoms [puckering parameters Q= 0.4579 (10) Å and φ=29.43 (13)°] whereas N2/C4/C6/C7/C10 is twisted about C6–C4 bond, [puckering parameters, Q= 0.3869 (10) Å and φ= 261.97 (15)°] adopting a half-chair conformation. The thiazolidine ring, S1/N2/C7–C9 is twisted about C8–S1 bond [puckering parameters, Q= 0.4519 (10) Å and φ= 333.87 (14)°] thereby, also adopting a half-chair conformations.

The mean plane of the dihydroacenaphthylene ring system [C10/C11/C25–C33, maximum deviation = 0.067 (1) Å at atom C10] makes dihedral angles of 28.31 (5) and 31.32 (6)°, respectively, with the two terminal benzene rings (C13–C18 & C19–C24).

An intramolecular O2—H1O2···N2 hydrogen bond (Table 1) forms an *S*(5) ring motif (Bernstein *et al.*, 1995). The bond lengths and angles are within normal ranges and comparable to the related structure (Kumar *et al.*, 2010*a*,*b*; Kumar *et al.*, 2011).

The crystal packing is shown in Fig. 2. The intermolecular C8—H8A···O1, C15—H15A···S1, C18—H18A···O1 and C24—H24A···O2 (Table 1) hydrogen bonds link the molecules into two-dimensional network parallel to *ac*-plane.

### Experimental

A mixture of 3,5-bis[(*E*)-phenylmethylidene] tetrahydro-4(1*H*)-pyridinone (1 mmol), acenaphthenequinone (1 mmol), and thiazolidine-2-carboxylic acid (1 mmol) were dissolved in methanol (5 ml) and refluxed for 1 h. After completion of the reaction as evident from TLC, the mixture was poured into water (50 ml). The precipitated solid was filtered and washed with water to obtain the product which was further purified by recrystallization from pet.ether-ethyl acetate mixture to obtain colourless blocks.

### Refinement

O-bound H atom was located from the difference map and refined freely, [O–H = 0.86 (2) Å]. The remaining H atoms were positioned geometrically [C–H = 0.95 and 1.00 Å] and refined using a riding model with *U*_iso_(H) = 1.2 *U*_eq_(C). The same *U^ij^* parameter was used for atom pairs C23/C24 and C22/C21.

### Crystal data

**Table:**

C_34_H_28_N_2_O_2_S	*F*(000) = 1112
*M**_r_* = 528.64	*D*_x_ = 1.328 Mg m^−^^3^
Monoclinic, *P*2_1_/*c*	Mo *K*α radiation, λ = 0.71073 Å
Hall symbol: -P 2ybc	Cell parameters from 9915 reflections
*a* = 11.2911 (1) Å	θ = 2.2–32.7°
*b* = 15.4317 (2) Å	µ = 0.16 mm^−^^1^
*c* = 15.1920 (2) Å	*T* = 100 K
β = 92.790 (1)°	Block, colourless
*V* = 2643.93 (5) Å^3^	0.45 × 0.41 × 0.31 mm
*Z* = 4	

### Data collection

**Table:**

Bruker SMART APEXII CCD diffractometer	9686 independent reflections
Radiation source: fine-focus sealed tube	8170 reflections with *I* > 2σ(*I*)
Graphite monochromator	*R*_int_ = 0.025
φ and ω scans	θ_max_ = 32.7°, θ_min_ = 2.6°
Absorption correction: multi-scan (*SADABS*; Bruker, 2009)	*h* = −17→17
*T*_min_ = 0.932, *T*_max_ = 0.953	*k* = −23→15
37231 measured reflections	*l* = −23→23

### Refinement

**Table:**

Refinement on *F*^2^	Primary atom site location: structure-invariant direct methods
Least-squares matrix: full	Secondary atom site location: difference Fourier map
*R*[*F*^2^ > 2σ(*F*^2^)] = 0.046	Hydrogen site location: inferred from neighbouring sites
*wR*(*F*^2^) = 0.129	H atoms treated by a mixture of independent and constrained refinement
*S* = 1.04	*w* = 1/[σ^2^(*F*_o_^2^) + (0.0652*P*)^2^ + 1.2561*P*] where *P* = (*F*_o_^2^ + 2*F*_c_^2^)/3
9686 reflections	(Δ/σ)_max_ = 0.001
344 parameters	Δρ_max_ = 1.18 e Å^−^^3^
0 restraints	Δρ_min_ = −1.12 e Å^−^^3^

### Special details

**Table:**

Experimental. The crystal was placed in the cold stream of an Oxford Cryosystems Cobra open-flow nitrogen cryostat (Cosier & Glazer, 1986) operating at 100.0 (1) K.
Geometry. All e.s.d.'s (except the e.s.d. in the dihedral angle between two l.s. planes) are estimated using the full covariance matrix. The cell e.s.d.'s are taken into account individually in the estimation of e.s.d.'s in distances, angles and torsion angles; correlations between e.s.d.'s in cell parameters are only used when they are defined by crystal symmetry. An approximate (isotropic) treatment of cell e.s.d.'s is used for estimating e.s.d.'s involving l.s. planes.
Refinement. Refinement of *F*^2^ against ALL reflections. The weighted *R*-factor *wR* and goodness of fit *S* are based on *F*^2^, conventional *R*-factors *R* are based on *F*, with *F* set to zero for negative *F*^2^. The threshold expression of *F*^2^ > σ(*F*^2^) is used only for calculating *R*-factors(gt) *etc*. and is not relevant to the choice of reflections for refinement. *R*-factors based on *F*^2^ are statistically about twice as large as those based on *F*, and *R*- factors based on ALL data will be even larger.

### Fractional atomic coordinates and isotropic or equivalent isotropic displacement parameters (Å^2^)

**Table:**

	*x*	*y*	*z*	*U*_iso_*/*U*_eq_	
S1	0.64487 (2)	0.10704 (2)	0.791294 (19)	0.02225 (7)	
O1	0.82800 (7)	0.32741 (5)	1.03565 (5)	0.01646 (15)	
O2	1.08336 (7)	0.06609 (5)	0.87449 (5)	0.01708 (15)	
N1	1.07824 (8)	0.13611 (6)	1.01109 (6)	0.01411 (15)	
N2	0.87576 (8)	0.13375 (6)	0.82898 (5)	0.01311 (15)	
C1	1.11599 (9)	0.21225 (7)	1.06386 (7)	0.01543 (18)	
H1A	1.1960	0.2297	1.0468	0.019*	
H1B	1.1224	0.1952	1.1267	0.019*	
C2	1.03363 (9)	0.29053 (7)	1.05418 (6)	0.01414 (17)	
C3	0.90690 (9)	0.27470 (7)	1.02482 (6)	0.01284 (16)	
C4	0.88395 (8)	0.18990 (6)	0.97700 (6)	0.01150 (16)	
C5	0.95369 (9)	0.11761 (7)	1.02825 (6)	0.01410 (17)	
H5A	0.9402	0.1207	1.0921	0.017*	
H5B	0.9301	0.0595	1.0060	0.017*	
C6	0.75412 (8)	0.17277 (7)	0.94872 (6)	0.01358 (17)	
H6A	0.7215	0.2275	0.9218	0.016*	
C7	0.76710 (9)	0.10734 (7)	0.87353 (7)	0.01441 (17)	
H7A	0.7781	0.0478	0.8986	0.017*	
C8	0.71970 (10)	0.18640 (9)	0.72578 (7)	0.0227 (2)	
H8A	0.6907	0.1839	0.6633	0.027*	
H8B	0.7080	0.2458	0.7485	0.027*	
C9	0.84945 (10)	0.15954 (7)	0.73615 (7)	0.01675 (18)	
H9A	0.8648	0.1104	0.6964	0.020*	
H9B	0.9010	0.2085	0.7204	0.020*	
C10	0.94831 (8)	0.19109 (6)	0.88798 (6)	0.01158 (16)	
C11	1.07541 (8)	0.14789 (7)	0.91425 (6)	0.01288 (16)	
C12	1.06869 (9)	0.37379 (7)	1.06055 (7)	0.01643 (18)	
H12A	1.0092	0.4165	1.0496	0.020*	
C13	1.18915 (10)	0.40547 (7)	1.08261 (7)	0.01790 (19)	
C14	1.26365 (11)	0.36572 (8)	1.14689 (8)	0.0229 (2)	
H14A	1.2363	0.3170	1.1782	0.027*	
C15	1.37756 (12)	0.39733 (10)	1.16505 (9)	0.0295 (3)	
H15A	1.4276	0.3701	1.2088	0.035*	
C16	1.41856 (12)	0.46848 (10)	1.11962 (10)	0.0319 (3)	
H16A	1.4971	0.4890	1.1314	0.038*	
C17	1.34442 (12)	0.50974 (9)	1.05683 (10)	0.0300 (3)	
H17A	1.3718	0.5590	1.0263	0.036*	
C18	1.23007 (11)	0.47867 (8)	1.03889 (9)	0.0236 (2)	
H18A	1.1793	0.5074	0.9966	0.028*	
C19	0.67082 (9)	0.14484 (8)	1.01828 (7)	0.01766 (19)	
C20	0.57348 (10)	0.19687 (9)	1.03556 (9)	0.0239 (2)	
H20A	0.5620	0.2500	1.0047	0.029*	
C21	0.49293 (15)	0.17194 (11)	1.09739 (13)	0.0437 (3)	
H21A	0.4269	0.2079	1.1084	0.052*	
C22	0.50910 (15)	0.09519 (11)	1.14248 (13)	0.0437 (3)	
H22A	0.4536	0.0778	1.1841	0.052*	
C23	0.60654 (14)	0.04303 (10)	1.12728 (11)	0.0371 (2)	
H23A	0.6186	−0.0092	1.1596	0.045*	
C24	0.68645 (15)	0.06729 (10)	1.06466 (11)	0.0371 (2)	
H24A	0.7519	0.0309	1.0535	0.045*	
C25	0.98054 (10)	0.27898 (7)	0.85111 (6)	0.01490 (17)	
C26	0.91211 (11)	0.34901 (7)	0.82466 (7)	0.0203 (2)	
H26A	0.8285	0.3478	0.8292	0.024*	
C27	0.96887 (15)	0.42305 (8)	0.79049 (8)	0.0287 (3)	
H27A	0.9220	0.4714	0.7717	0.034*	
C28	1.08990 (15)	0.42668 (8)	0.78384 (8)	0.0307 (3)	
H28A	1.1247	0.4764	0.7588	0.037*	
C29	1.16320 (12)	0.35705 (8)	0.81394 (8)	0.0248 (2)	
C30	1.28876 (13)	0.35283 (10)	0.81486 (9)	0.0326 (3)	
H30A	1.3324	0.4002	0.7930	0.039*	
C31	1.34778 (12)	0.28063 (11)	0.84717 (9)	0.0328 (3)	
H31A	1.4318	0.2791	0.8463	0.039*	
C32	1.28735 (10)	0.20819 (9)	0.88192 (8)	0.0243 (2)	
H32A	1.3300	0.1594	0.9047	0.029*	
C33	1.16550 (9)	0.21092 (7)	0.88160 (7)	0.01648 (18)	
C34	1.10499 (10)	0.28405 (7)	0.84706 (7)	0.01705 (19)	
H1O2	1.0163 (18)	0.0606 (13)	0.8458 (13)	0.037 (5)*	

### Atomic displacement parameters (Å^2^)

**Table:**

	*U*^11^	*U*^22^	*U*^33^	*U*^12^	*U*^13^	*U*^23^
S1	0.01626 (12)	0.03024 (16)	0.01985 (13)	−0.00226 (10)	−0.00317 (9)	−0.00495 (11)
O1	0.0178 (3)	0.0163 (3)	0.0153 (3)	0.0038 (3)	0.0011 (3)	−0.0018 (3)
O2	0.0178 (3)	0.0134 (3)	0.0199 (3)	0.0030 (3)	−0.0001 (3)	−0.0043 (3)
N1	0.0145 (3)	0.0147 (4)	0.0130 (3)	0.0011 (3)	−0.0001 (3)	0.0010 (3)
N2	0.0152 (3)	0.0138 (4)	0.0102 (3)	−0.0011 (3)	0.0004 (3)	0.0001 (3)
C1	0.0167 (4)	0.0163 (4)	0.0130 (4)	0.0018 (3)	−0.0019 (3)	−0.0004 (3)
C2	0.0160 (4)	0.0155 (4)	0.0109 (4)	0.0012 (3)	0.0005 (3)	−0.0008 (3)
C3	0.0158 (4)	0.0135 (4)	0.0092 (3)	0.0009 (3)	0.0008 (3)	0.0007 (3)
C4	0.0132 (4)	0.0113 (4)	0.0101 (3)	0.0010 (3)	0.0012 (3)	0.0010 (3)
C5	0.0161 (4)	0.0135 (4)	0.0127 (4)	0.0012 (3)	0.0014 (3)	0.0037 (3)
C6	0.0131 (4)	0.0139 (4)	0.0137 (4)	0.0008 (3)	0.0008 (3)	0.0002 (3)
C7	0.0141 (4)	0.0150 (4)	0.0141 (4)	−0.0011 (3)	0.0008 (3)	−0.0008 (3)
C8	0.0236 (5)	0.0274 (6)	0.0166 (4)	0.0053 (4)	−0.0042 (4)	0.0018 (4)
C9	0.0213 (4)	0.0184 (5)	0.0104 (4)	0.0016 (4)	−0.0005 (3)	0.0004 (3)
C10	0.0142 (4)	0.0100 (4)	0.0107 (4)	0.0005 (3)	0.0010 (3)	0.0008 (3)
C11	0.0138 (4)	0.0123 (4)	0.0126 (4)	0.0009 (3)	0.0011 (3)	−0.0005 (3)
C12	0.0169 (4)	0.0162 (4)	0.0161 (4)	0.0010 (4)	0.0006 (3)	−0.0019 (4)
C13	0.0181 (4)	0.0170 (5)	0.0186 (4)	−0.0004 (4)	0.0002 (3)	−0.0034 (4)
C14	0.0250 (5)	0.0230 (5)	0.0201 (5)	−0.0039 (4)	−0.0055 (4)	−0.0011 (4)
C15	0.0257 (6)	0.0306 (7)	0.0309 (6)	−0.0037 (5)	−0.0110 (5)	−0.0013 (5)
C16	0.0223 (5)	0.0316 (7)	0.0411 (7)	−0.0075 (5)	−0.0057 (5)	−0.0033 (6)
C17	0.0252 (6)	0.0250 (6)	0.0397 (7)	−0.0075 (5)	−0.0003 (5)	0.0024 (5)
C18	0.0215 (5)	0.0187 (5)	0.0303 (6)	−0.0014 (4)	−0.0015 (4)	0.0022 (4)
C19	0.0151 (4)	0.0200 (5)	0.0182 (4)	−0.0011 (4)	0.0048 (3)	−0.0026 (4)
C20	0.0164 (4)	0.0243 (5)	0.0313 (6)	0.0003 (4)	0.0055 (4)	−0.0074 (5)
C21	0.0380 (5)	0.0378 (6)	0.0585 (7)	−0.0036 (5)	0.0341 (5)	−0.0087 (5)
C22	0.0380 (5)	0.0378 (6)	0.0585 (7)	−0.0036 (5)	0.0341 (5)	−0.0087 (5)
C23	0.0419 (6)	0.0308 (5)	0.0411 (5)	0.0034 (4)	0.0271 (5)	0.0101 (4)
C24	0.0419 (6)	0.0308 (5)	0.0411 (5)	0.0034 (4)	0.0271 (5)	0.0101 (4)
C25	0.0229 (4)	0.0109 (4)	0.0112 (4)	−0.0011 (3)	0.0034 (3)	0.0004 (3)
C26	0.0339 (6)	0.0132 (4)	0.0143 (4)	0.0035 (4)	0.0043 (4)	0.0025 (4)
C27	0.0562 (8)	0.0124 (5)	0.0183 (5)	0.0024 (5)	0.0106 (5)	0.0031 (4)
C28	0.0574 (9)	0.0147 (5)	0.0212 (5)	−0.0100 (5)	0.0148 (5)	0.0009 (4)
C29	0.0382 (6)	0.0201 (5)	0.0169 (5)	−0.0129 (5)	0.0103 (4)	−0.0033 (4)
C30	0.0378 (7)	0.0359 (7)	0.0252 (6)	−0.0224 (6)	0.0142 (5)	−0.0062 (5)
C31	0.0235 (5)	0.0487 (9)	0.0271 (6)	−0.0174 (6)	0.0101 (5)	−0.0098 (6)
C32	0.0169 (4)	0.0349 (7)	0.0213 (5)	−0.0056 (4)	0.0048 (4)	−0.0065 (5)
C33	0.0169 (4)	0.0192 (5)	0.0137 (4)	−0.0037 (4)	0.0040 (3)	−0.0030 (3)
C34	0.0238 (5)	0.0152 (4)	0.0127 (4)	−0.0053 (4)	0.0061 (3)	−0.0020 (3)

### Geometric parameters (Å, º)

**Table:**

S1—C8	1.8126 (13)	C14—C15	1.3906 (17)
S1—C7	1.8159 (10)	C14—H14A	0.9500
O1—C3	1.2234 (12)	C15—C16	1.388 (2)
O2—C11	1.4042 (12)	C15—H15A	0.9500
O2—H1O2	0.86 (2)	C16—C17	1.392 (2)
N1—C5	1.4707 (13)	C16—H16A	0.9500
N1—C1	1.4735 (14)	C17—C18	1.3917 (17)
N1—C11	1.4812 (13)	C17—H17A	0.9500
N2—C10	1.4784 (13)	C18—H18A	0.9500
N2—C9	1.4815 (13)	C19—C24	1.3956 (19)
N2—C7	1.4867 (13)	C19—C20	1.3962 (15)
C1—C2	1.5272 (15)	C20—C21	1.3931 (18)
C1—H1A	0.9900	C20—H20A	0.9500
C1—H1B	0.9900	C21—C22	1.376 (3)
C2—C12	1.3465 (15)	C21—H21A	0.9500
C2—C3	1.4980 (14)	C22—C23	1.392 (2)
C3—C4	1.5130 (14)	C22—H22A	0.9500
C4—C6	1.5304 (14)	C23—C24	1.3938 (18)
C4—C5	1.5527 (14)	C23—H23A	0.9500
C4—C10	1.5663 (13)	C24—H24A	0.9500
C5—H5A	0.9900	C25—C26	1.3770 (15)
C5—H5B	0.9900	C25—C34	1.4117 (15)
C6—C19	1.5114 (14)	C26—C27	1.4206 (17)
C6—C7	1.5369 (14)	C26—H26A	0.9500
C6—H6A	1.0000	C27—C28	1.376 (2)
C7—H7A	1.0000	C27—H27A	0.9500
C8—C9	1.5233 (16)	C28—C29	1.418 (2)
C8—H8A	0.9900	C28—H28A	0.9500
C8—H8B	0.9900	C29—C34	1.4093 (15)
C9—H9A	0.9900	C29—C30	1.419 (2)
C9—H9B	0.9900	C30—C31	1.376 (2)
C10—C25	1.5183 (14)	C30—H30A	0.9500
C10—C11	1.6147 (14)	C31—C32	1.4244 (19)
C11—C33	1.5086 (14)	C31—H31A	0.9500
C12—C13	1.4686 (15)	C32—C33	1.3763 (15)
C12—H12A	0.9500	C32—H32A	0.9500
C13—C14	1.3992 (16)	C33—C34	1.4074 (16)
C13—C18	1.4001 (17)		
			
C8—S1—C7	90.98 (5)	C2—C12—H12A	116.6
C11—O2—H1O2	103.5 (14)	C13—C12—H12A	116.6
C5—N1—C1	108.19 (8)	C14—C13—C18	118.93 (11)
C5—N1—C11	103.02 (8)	C14—C13—C12	122.13 (11)
C1—N1—C11	115.73 (8)	C18—C13—C12	118.93 (10)
C10—N2—C9	119.72 (8)	C15—C14—C13	120.23 (12)
C10—N2—C7	109.47 (7)	C15—C14—H14A	119.9
C9—N2—C7	112.04 (8)	C13—C14—H14A	119.9
N1—C1—C2	114.81 (8)	C16—C15—C14	120.42 (12)
N1—C1—H1A	108.6	C16—C15—H15A	119.8
C2—C1—H1A	108.6	C14—C15—H15A	119.8
N1—C1—H1B	108.6	C15—C16—C17	119.90 (12)
C2—C1—H1B	108.6	C15—C16—H16A	120.1
H1A—C1—H1B	107.5	C17—C16—H16A	120.1
C12—C2—C3	116.76 (9)	C18—C17—C16	119.85 (13)
C12—C2—C1	124.91 (9)	C18—C17—H17A	120.1
C3—C2—C1	117.91 (9)	C16—C17—H17A	120.1
O1—C3—C2	122.88 (9)	C17—C18—C13	120.63 (12)
O1—C3—C4	122.07 (9)	C17—C18—H18A	119.7
C2—C3—C4	115.01 (8)	C13—C18—H18A	119.7
C3—C4—C6	115.06 (8)	C24—C19—C20	118.66 (11)
C3—C4—C5	108.05 (8)	C24—C19—C6	121.99 (10)
C6—C4—C5	118.17 (8)	C20—C19—C6	119.33 (11)
C3—C4—C10	109.18 (8)	C21—C20—C19	120.91 (14)
C6—C4—C10	103.77 (7)	C21—C20—H20A	119.5
C5—C4—C10	101.31 (7)	C19—C20—H20A	119.5
N1—C5—C4	103.75 (8)	C22—C21—C20	119.88 (14)
N1—C5—H5A	111.0	C22—C21—H21A	120.1
C4—C5—H5A	111.0	C20—C21—H21A	120.1
N1—C5—H5B	111.0	C21—C22—C23	120.14 (13)
C4—C5—H5B	111.0	C21—C22—H22A	119.9
H5A—C5—H5B	109.0	C23—C22—H22A	119.9
C19—C6—C4	118.24 (8)	C22—C23—C24	120.08 (15)
C19—C6—C7	114.68 (9)	C22—C23—H23A	120.0
C4—C6—C7	101.39 (8)	C24—C23—H23A	120.0
C19—C6—H6A	107.3	C23—C24—C19	120.31 (13)
C4—C6—H6A	107.3	C23—C24—H24A	119.8
C7—C6—H6A	107.3	C19—C24—H24A	119.8
N2—C7—C6	105.60 (8)	C26—C25—C34	119.30 (10)
N2—C7—S1	107.70 (7)	C26—C25—C10	131.87 (10)
C6—C7—S1	114.60 (7)	C34—C25—C10	108.82 (9)
N2—C7—H7A	109.6	C25—C26—C27	118.75 (12)
C6—C7—H7A	109.6	C25—C26—H26A	120.6
S1—C7—H7A	109.6	C27—C26—H26A	120.6
C9—C8—S1	103.36 (8)	C28—C27—C26	121.79 (12)
C9—C8—H8A	111.1	C28—C27—H27A	119.1
S1—C8—H8A	111.1	C26—C27—H27A	119.1
C9—C8—H8B	111.1	C27—C28—C29	120.75 (11)
S1—C8—H8B	111.1	C27—C28—H28A	119.6
H8A—C8—H8B	109.1	C29—C28—H28A	119.6
N2—C9—C8	108.59 (8)	C34—C29—C28	116.47 (12)
N2—C9—H9A	110.0	C34—C29—C30	116.29 (13)
C8—C9—H9A	110.0	C28—C29—C30	127.24 (12)
N2—C9—H9B	110.0	C31—C30—C29	120.46 (12)
C8—C9—H9B	110.0	C31—C30—H30A	119.8
H9A—C9—H9B	108.4	C29—C30—H30A	119.8
N2—C10—C25	116.53 (8)	C30—C31—C32	122.35 (12)
N2—C10—C4	104.46 (7)	C30—C31—H31A	118.8
C25—C10—C4	117.30 (8)	C32—C31—H31A	118.8
N2—C10—C11	111.24 (8)	C33—C32—C31	118.13 (13)
C25—C10—C11	103.53 (8)	C33—C32—H32A	120.9
C4—C10—C11	103.05 (7)	C31—C32—H32A	120.9
O2—C11—N1	108.55 (8)	C32—C33—C34	119.53 (11)
O2—C11—C33	112.33 (8)	C32—C33—C11	131.99 (11)
N1—C11—C33	115.05 (8)	C34—C33—C11	108.47 (9)
O2—C11—C10	109.85 (8)	C33—C34—C29	123.21 (11)
N1—C11—C10	105.79 (7)	C33—C34—C25	113.91 (9)
C33—C11—C10	104.94 (8)	C29—C34—C25	122.84 (11)
C2—C12—C13	126.86 (10)		
			
C5—N1—C1—C2	−50.94 (11)	C4—C10—C11—N1	−6.12 (10)
C11—N1—C1—C2	64.00 (11)	N2—C10—C11—C33	120.40 (8)
N1—C1—C2—C12	−148.69 (10)	C25—C10—C11—C33	−5.50 (9)
N1—C1—C2—C3	23.57 (13)	C4—C10—C11—C33	−128.18 (8)
C12—C2—C3—O1	−25.83 (14)	C3—C2—C12—C13	−176.07 (10)
C1—C2—C3—O1	161.28 (9)	C1—C2—C12—C13	−3.73 (17)
C12—C2—C3—C4	151.68 (9)	C2—C12—C13—C14	−39.53 (17)
C1—C2—C3—C4	−21.21 (12)	C2—C12—C13—C18	141.52 (12)
O1—C3—C4—C6	−3.72 (13)	C18—C13—C14—C15	−1.72 (18)
C2—C3—C4—C6	178.74 (8)	C12—C13—C14—C15	179.33 (12)
O1—C3—C4—C5	−138.19 (9)	C13—C14—C15—C16	−0.1 (2)
C2—C3—C4—C5	44.28 (10)	C14—C15—C16—C17	1.4 (2)
O1—C3—C4—C10	112.43 (10)	C15—C16—C17—C18	−1.0 (2)
C2—C3—C4—C10	−65.10 (10)	C16—C17—C18—C13	−0.8 (2)
C1—N1—C5—C4	74.41 (9)	C14—C13—C18—C17	2.17 (19)
C11—N1—C5—C4	−48.61 (9)	C12—C13—C18—C17	−178.84 (12)
C3—C4—C5—N1	−71.09 (9)	C4—C6—C19—C24	−62.92 (16)
C6—C4—C5—N1	156.08 (8)	C7—C6—C19—C24	56.65 (15)
C10—C4—C5—N1	43.59 (9)	C4—C6—C19—C20	118.64 (11)
C3—C4—C6—C19	−76.10 (11)	C7—C6—C19—C20	−121.79 (11)
C5—C4—C6—C19	53.56 (12)	C24—C19—C20—C21	−0.4 (2)
C10—C4—C6—C19	164.69 (9)	C6—C19—C20—C21	178.11 (13)
C3—C4—C6—C7	157.62 (8)	C19—C20—C21—C22	0.2 (3)
C5—C4—C6—C7	−72.71 (10)	C20—C21—C22—C23	0.8 (3)
C10—C4—C6—C7	38.41 (9)	C21—C22—C23—C24	−1.7 (3)
C10—N2—C7—C6	18.07 (10)	C22—C23—C24—C19	1.4 (3)
C9—N2—C7—C6	−117.21 (9)	C20—C19—C24—C23	−0.4 (2)
C10—N2—C7—S1	140.93 (7)	C6—C19—C24—C23	−178.88 (14)
C9—N2—C7—S1	5.65 (10)	N2—C10—C25—C26	64.02 (14)
C19—C6—C7—N2	−163.63 (8)	C4—C10—C25—C26	−60.86 (15)
C4—C6—C7—N2	−35.04 (9)	C11—C10—C25—C26	−173.53 (11)
C19—C6—C7—S1	78.03 (10)	N2—C10—C25—C34	−117.11 (9)
C4—C6—C7—S1	−153.39 (7)	C4—C10—C25—C34	118.01 (9)
C8—S1—C7—N2	−25.83 (8)	C11—C10—C25—C34	5.34 (10)
C8—S1—C7—C6	91.32 (8)	C34—C25—C26—C27	2.64 (16)
C7—S1—C8—C9	37.74 (8)	C10—C25—C26—C27	−178.58 (10)
C10—N2—C9—C8	−107.21 (10)	C25—C26—C27—C28	−0.48 (18)
C7—N2—C9—C8	22.98 (12)	C26—C27—C28—C29	−2.16 (19)
S1—C8—C9—N2	−40.56 (10)	C27—C28—C29—C34	2.45 (18)
C9—N2—C10—C25	6.48 (13)	C27—C28—C29—C30	−177.20 (12)
C7—N2—C10—C25	−124.83 (9)	C34—C29—C30—C31	−0.30 (18)
C9—N2—C10—C4	137.64 (9)	C28—C29—C30—C31	179.35 (13)
C7—N2—C10—C4	6.32 (10)	C29—C30—C31—C32	−0.9 (2)
C9—N2—C10—C11	−111.85 (9)	C30—C31—C32—C33	0.93 (19)
C7—N2—C10—C11	116.84 (8)	C31—C32—C33—C34	0.29 (16)
C3—C4—C10—N2	−151.48 (8)	C31—C32—C33—C11	−178.52 (11)
C6—C4—C10—N2	−28.32 (9)	O2—C11—C33—C32	−57.92 (15)
C5—C4—C10—N2	94.68 (8)	N1—C11—C33—C32	66.94 (15)
C3—C4—C10—C25	−20.77 (11)	C10—C11—C33—C32	−177.23 (11)
C6—C4—C10—C25	102.38 (10)	O2—C11—C33—C34	123.18 (9)
C5—C4—C10—C25	−134.61 (9)	N1—C11—C33—C34	−111.96 (10)
C3—C4—C10—C11	92.17 (9)	C10—C11—C33—C34	3.87 (10)
C6—C4—C10—C11	−144.67 (8)	C32—C33—C34—C29	−1.56 (16)
C5—C4—C10—C11	−21.66 (9)	C11—C33—C34—C29	177.50 (10)
C5—N1—C11—O2	−84.52 (9)	C32—C33—C34—C25	−179.63 (10)
C1—N1—C11—O2	157.64 (8)	C11—C33—C34—C25	−0.57 (12)
C5—N1—C11—C33	148.67 (9)	C28—C29—C34—C33	−178.14 (10)
C1—N1—C11—C33	30.82 (12)	C30—C29—C34—C33	1.55 (16)
C5—N1—C11—C10	33.32 (10)	C28—C29—C34—C25	−0.24 (16)
C1—N1—C11—C10	−84.52 (9)	C30—C29—C34—C25	179.45 (10)
N2—C10—C11—O2	−0.56 (10)	C26—C25—C34—C33	175.75 (9)
C25—C10—C11—O2	−126.46 (8)	C10—C25—C34—C33	−3.29 (12)
C4—C10—C11—O2	110.86 (8)	C26—C25—C34—C29	−2.33 (16)
N2—C10—C11—N1	−117.53 (8)	C10—C25—C34—C29	178.64 (9)
C25—C10—C11—N1	116.56 (8)		

### Hydrogen-bond geometry (Å, º)

**Table:**

*D*—H···*A*	*D*—H	H···*A*	*D*···*A*	*D*—H···*A*
O2—H1*O*2···N2	0.86 (2)	1.95 (2)	2.6277 (12)	134.4 (18)
C8—H8*A*···O1^i^	0.99	2.55	3.1981 (14)	123
C15—H15*A*···S1^ii^	0.95	2.72	3.4970 (13)	139
C18—H18*A*···O1^iii^	0.95	2.60	3.2550 (15)	127
C24—H24*A*···O2^iv^	0.95	2.59	3.4077 (18)	145

Symmetry codes: (i) *x*, −*y*+1/2, *z*−1/2; (ii) *x*+1, −*y*+1/2, *z*+1/2; (iii) −*x*+2, −*y*+1, −*z*+2; (iv) −*x*+2, −*y*, −*z*+2.
